# Measuring spatial accessibility to urban services for older adults: an application to healthcare facilities in Milan

**DOI:** 10.1186/s12544-022-00544-3

**Published:** 2022-06-02

**Authors:** Carmen Guida, Gerardo Carpentieri, Houshmand Masoumi

**Affiliations:** 1 Department of Civil, Architectural and Environmental Engineering, University of Naples Federico II, Naples, Italy; 2 Center for Technology and Society, Technische Universität Berlin, Berlin, Germany

**Keywords:** City, Accessibility, Elderly people, Land-use, Transit, Health care facilities

## Abstract

This study proposes a Geographic Information Systems-based methodology to measure accessibility to urban services from the elderly perspective to support urban planning processes. Specifically, it seeks to understand and clarify how the urban environment can influence the quality of life for older adults, mostly through pedestrian and public transport networks, locations of essential urban services, and the organisation of their resources. In light of a significant demographic change, policymakers will have to promote age-friendly urban planning approaches to guarantee equal access to services and activities. We propose a methodology to measure accessibility to healthcare provision services that considers land-use and mobility features and older adults' behavioural traits. The method belongs to the family of 2SFCA—2 steps floating catchment area—which evaluate accessibility as the combination of both supply and demand of urban services. Therefore, we have introduced innovative elements to shape actual mobility opportunities for the elderly and their travel choices. The methodology was applied to Milan's city to measure accessibility to the Health Protection Agency (ATS) services, which is responsible for healthcare assistance to the elderly dwelling in the city. The outputs show that a significant share of older people (almost 40%) suffer from poor accessibility to primary health services and that they mostly live in the city periphery. Moreover, the application to a case study has shown that the methodology could identify the critical aspects needed to aid urban planning to achieve a high quality of life for elderly people.

## Introduction

In 2020, the global population aged 60 years and over was just over 1 billion, representing 13.5% of the world’s population of 7.8 billion. That number is 2.5 times greater than in 1980 (382 million) and is projected to reach nearly 2.1 billion by 2050 (United Nations, 2020). This phenomenon has very different implications according to one’s perspective; in fact, it is much more evident in developed countries than in developing nations. That means that the European population is much older than in other parts of the world and that on average, Europeans enjoy longer lives with better health [1]. Life expectancy at birth has increased by approximately 10 years for both men and women over the last 5 decades. At the beginning of 2020, the pandemic outbreak in Europe has stressed the vulnerabilities of an ageing population but is not thought likely to have changed this overall positive trend on life expectancy. In 2070, life expectancy at birth is projected to reach 86.1 years for men, up from 78.2 in 2018. For women, it is estimated at 90.3—up from 83.7. Where you live has a significant influence on your life expectancy. At the national level, life expectancy at birth ranges from 83.5 in Spain to 75 in Bulgaria. It is worth bearing in mind that meanwhile, due to different economic and social issues, the average number of childbirths per woman in Europe have significantly decreased since 1960. It recovered somewhat in the 2000s and then roughly stabilised in the decade that followed. The combination of these two phenomena has increased the number and share of people in the older age groups, while the working-age population (20–64 years) is projected to decrease (from 59% nowadays to 51% in 2070). Hence, the role and contribution of elderly people to families, communities, and society's social, political, and economic well-being will be even greater than it is today.

On the other hand, ageing is associated with an increased vulnerability and dependence on medical care services [2]. This is combined with the occurrence of social isolation, loneliness, and depression due to age-associated limitations that influence mobility.

From the perspective of social equity, everyone should have the opportunity to access essential services and opportunities equally, especially people who need them the most to maintain a good quality of life. Adequate accessibility to healthcare service is one of the vital elements for holding an advanced society status. World Health Organization under the human right concept describe accessibility as availability of health services within a safe and reasonable physical reach to all section of the population especially vulnerable and marginal groups likely ethnic minorities, women, children, aged groups and persons with disabilities [3].

With respect to these observations, more age-friendly approaches are needed in our cities, and it would be a challenge to prepare for these developments in such a way that both current and future generations of older people can benefit from age-friendly urban planning strategies. Although many academic studies developed to assess urban accessibility to healthcare facilities and other essential urban services, their full implementation in urban planning practices is still missing [4]. Our research work aims to contribute both to the scientific debate, by improving existing methodologies for measuring accessibility, with a special focus on the elderly population, and to the integration of GIS-based decision support methodologies into spatial government practice.

Our contribution is divided into five parts. Following this introduction, a literature review of urban accessibility definition is provided. In Sect. 3, we propose a GIS-based procedure in order to compute the urban accessibility in urban areas; in Sect. 4, we discuss the application of the methodology to the city of Milan; in Sect. 5, the results are presented and discussed.

### Urban accessibility: the state of research

Accessibility to physical environments, transport systems, information and communication technologies and other facilities and services open to the public [5] represents an essential right to live independently and participate fully in all spheres of life, according to Article 9 of United Nations Convention on the Rights of Persons with Disabilities (2007).

This right can be translated into the concept of mobility capital [6], which has found plenty of agreement in both academic and professional fields. It considers available resources and restrictions on access to services and products. According to this approach, mobility capital considers mobility as a means and a resource for action, available to users to satisfy their needs and achieve their goals. Just as much as other capitals, mobility capital is heterogeneously spread in society and, hence, a lack of resources and capabilities represents a potential form of social and spatial exclusion [7].

For many years, policymakers and relevant actors in urban and territorial governance studied the urban accessibility issue as a transport-related problem rather than a multidisciplinary topic that incorporates localisation and distribution of opportunities and resources within an urban area [8]. As a result, some urban planning practices, such as transit-oriented developments as well as walkability policies indirectly affect the perceived accessibility but a more holistic approach to the issue is still missing [9].

The urban accessibility paradigm is a complex and multidisciplinary concept which may have led to a noticeable gap between the academic findings and their usefulness in real-world planning practice [10]. That was mostly due to difficulties in computing and introducing accessibility measures in decision-making practices. The advent of Geographical Information Systems (GIS) has made much more practical the development of accessibility-oriented planning tools, and many commercial packages are now available.

This study proposes a GIS-based procedure to evaluate urban accessibility to primary healthcare services for elderly people and to support decision-makers in identifying spatial and organization problems to better allocate resources in local welfare-policy restructuring.

In recent decades, several accessibility measures have been developed in an attempt to consider different physical, social, physiological, and economic issues [11]. According to the literature [12–15], four components can explain accessibility and its lack of homogeneity within an urban environment:

- the land-use component, which concerns both demand characteristics, such as people origin locations, and supply system features (activities, jobs, services, etc. within the study area) [16],
- the transportation component, which is the combination of both supply (network infrastructures and generalised costs) and demand (passengers or freight) [17],
- the individual component that considers people’s needs, abilities and opportunities (annual income, age, household car-ownership, etc.) [18],
- the temporal component, which is useful in matching transport and activity schedules to the individuals’ available time for participate in certain activities [19].

**Fig. 1:**
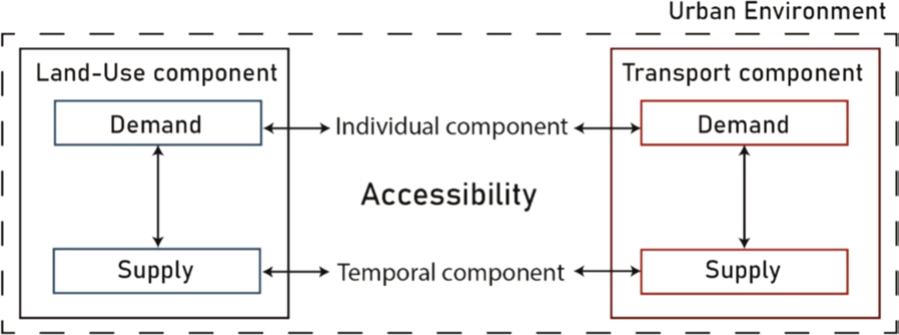
Accessibility components (authors’ elaboration)

Figure 1, below, represents accessibility components and their interrelations. Both the Land-Use and the Transport components are made of a supply and demand side. The level of accessibility cannot disregard the balance between these two issues. In particular, the supply component shall comprise the number and level of service of activities spread within an urban system, including public transport offer and walkable infrastructures (for the transport system). For what concerns the demand side it is made, for both components, of city dwellers and their mobility, social and economic capital. Hence, the individual component is an essential trait to better shape the needs and socio-economic potentials of urban citizens. Indeed, the temporal component is essential to model the variable availability and distribution of services and their resources as climate seasons change (for instance), as well as during the span of a day.

It is worth noting that, according to this scheme, accessibility measures have to account for several complex urban phenomena, such as demand-side factors, population distribution patterns, behavioural aspects, and individual capabilities [20, 21]. The two-step floating catchment area (2SFCA) models, from the family of gravity-based measures, are more recent and complex approaches and have the advantage of considering these features as well as their interactions. 2SFCA measures were built upon the concept of ‘catchment area’ [22]. Their models collect several urban components necessary for conceptualising accessibility (including supply, demand, interaction, and competition into a two-step procedure. The first step aims to evaluate the ratio between the supply (number of resources per each facility and the demand (number of potential users, quantifying the stress level of services, the second step estimates accessibility as the sum of available services, weighted by their ratios and their distances from users.

We propose a new methodology to measure urban accessibility to primary healthcare services for the elderly, considering their behaviour and mobility capital and, hence, suggesting potential interventions and scenarios to improve accessibility and consequently, the quality of life of this vulnerable group of people. Hence, our research aims at answering the following questions: (1) how to measure spatial accessibility to services, taking into account demand and supply-side features, individual limits and behaviours; (2) how to turn scientific-based knowledge into real-world practice.

According to the transport-oriented approach, usually applied to these studies, accessibility can be measured as road and transit capacity, travel frequency, and level of service [23], which cannot consider the whole complexity of urban systems.

During the last century, the widespread use of new mobility systems, mainly due to the increasing use of private cars, completely upset urban environments [24, 25]. Furthermore, financial and economic events, global capitalism, and the rise of the Internet increased the sprawl of activities and people on a wide urban territory [26–28], despite initially-innocent predictions. Consequently, although some services tend to keep a proximity attribute, such as educational systems and infant care [29–31], privatisation processes, rationalisation, and relocation tend to drop an even higher number of activities from residences: family-based corner shops are replaced by great distribution structures, places out of municipality boundaries are becoming distribution spaces of productive units; shopping and leisure centres. Given these significant topics, accessibility paradigms need broader thinking on urban issues, opportunities and possibilities for travel, activities and services spread within an urban environment, and individual limits and capabilities [32].

## Method and materials

We proposed an Elderly-oriented Multimodal Two-Step Floating Catchment Area (EM2SFCA) method, including additional variables and steps to be undertaken before the original two-step procedure of 2SFCA. This methodology will measure accessibility to urban services for a static scenario, in order to evaluate spatial accessibility to urban services from the elderly perspective, during an ordinary working day [33]. Concerning this issue, we introduced some further variables to consider the actual mobility capital for people aged 65 and over [34, 35]. In comparison to a first application of the methodology, we introduced variable distance decay functions to better model mobility capital of older adults, while ageing. Moreover, by validating the method with a different urban context, we proved that multiple management strategies of activity systems may deeply influence accessibility perception.

**Fig. 2:**
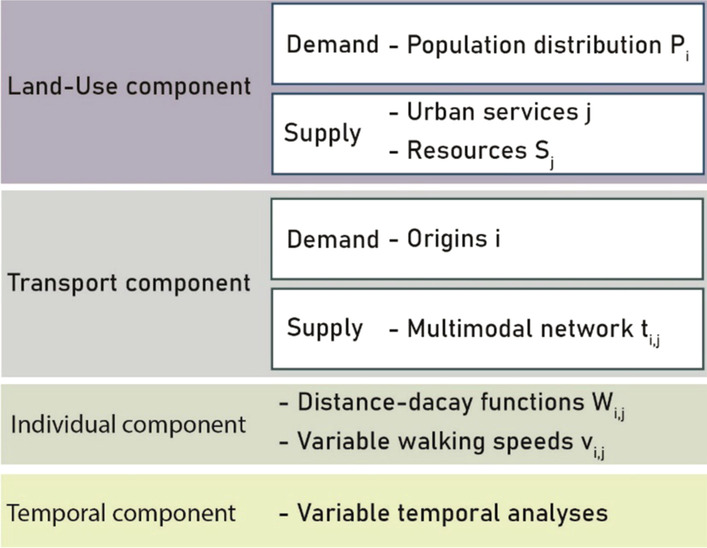
EM2SFCA input (authors’ elaboration)

Hence, we developed a methodology based in a Geographical Information System (GIS) environment to take advantage of storing, analysing, and visualising capabilities of these technologies so that it could be easily replied in different contexts [36]. Moreover, to guarantee strong theoretical bases, we have taken separately into account each accessibility component, as described in Fig. 2.

Figure 2 sums the input data for the EM2SFCA model, according to the accessibility component described above. As concerns the land-use and transport components, both supply and demand-side features were considered, since the level of accessibility in an urban environment, meaning the number of available resources, depends on their offer and demand interactions.

Starting from land use, the demand for healthcare provisions consists of population distribution P_i_. *i* refers to the smallest territorial unit, a 50-m-side hexagonal cell [37]. The use of hexagonal grid (rather than a square one or census track) is recommended for dealing with problems related to the connectivity of different space units and identifying shorter paths for calculating travel distances [38]. On the supply-side, the land-use component is described by the combination of healthcare structures (*j*) locations and their resources—*S*_*j*_—described, for instance, by the number of working physicians, available beds or surface of buildings (mq) [39].

Concerning the transport component, population distribution, and residential locations of dwellers, all influence demand and hence, were included in the EM2SFCA model. The latter consists of barycentre of hexagonal cells—*i*—approximately considered as origins of travel journeys to reach healthcare facilities. We developed a multimodal network for time travel analyses comprised of walkable roads and transit (bus and metro) lines on the supply-side. This essential and innovative issue in academic research depends on some significant considerations: elderly people are highly dependent on public transport, as they have relatively limited use of private cars and, more generally, of mobility capital.

Dealing with this issue, the individual component is essential in assessing accessibility in the urban environment from the elderly perspective. For this reason, we attributed different walking speeds (v_i,j_) to the links of pedestrian network and introduced Gaussian distance-decay functions—*W*_*i,j*_—[40], one per age group (65–69, 70–74; ≥ 75), to diversify travel times.

1 $$W_{ij} = e^{{{\raise0.7ex\hbox{${ - t_{ij}^{2} }$} \!\mathord{\left/ {\vphantom {{ - t_{ij}^{2} } \beta }}\right.\kern-\nulldelimiterspace} \!\lower0.7ex\hbox{$\beta $}}}}$$

According to two studies [41, 42], we introduced decreasing walking speed for each age group: 0.8 m/s for those aged 65–69,0.7 m/s for people aged between 70 and 74; 0.6 m/s for the oldest (≥ 75).

As far as for the Gaussian distance-decay functions, their main characteristic is that they quickly decrease when time travel increases and gets close to the maximum time that each elderly age category manages to hold (according to their physical capabilities) to access health services. Moreover, as people age, their mobility capital decreases; hence, β coefficients were set equal to 180 for people aged between 65 and 69, 160 for those between 70–74 and 140 for those aged 75 and over, to best represent mobility attitudes of different elderly age categories according to outcomes in the scientific literature [43].

The last component relates to the variable accessibility perceived by dwellers due to temporal issues. The transport network model is based on GTFS (General Transit Feed Specification) data to run separate temporal analyses and evaluate accessibility variations during a weekday. For our application, we run accessibility analyses just for one temporal scenario: 9 a.m. for an ordinary weekday. This temporal slot has been chosen to provide accessibility assessments for a peak hour, both for transport and service components.

The inputted data described above are essential features to process the EM2SFCA model, which consists of two consequent steps, described by the following formulas.

2 $$R_{j} = \frac{{S_{j} }}{{\mathop \sum \nolimits_{age - group} \mathop \sum \nolimits_{i} P_{i} \cdot W_{ij}^{age - group9} }}$$

3 $$A_{i} = \mathop \sum \limits_{j} \mathop \sum \limits_{age - group} R_{j} \cdot W_{ij}^{age - group}$$

*R*_*j*_ is the supply–demand ratio of each healthcare structure, while *A*_*i*_ refers to the overall accessibility to each *i* hexagonal cell’s primary healthcare provision.

For the representation of results by the GIS in maps and tables, the proposed method implements a quantile classification of measured accessibility in ten levels, from level 1 (low accessibility) to level 10 (high accessibility). The quantile classification is defined as cumulated accessibility values considering the three age categories [44]. This representation is based on solid scientific and practical ground [45] since it provides a better spatial representation of methodological results and suggests to policy-makers priority areas for action and intervention.

## Case study

The case study we applied to the developed methodology is Milan, the second most populated city in Italy after Rome, with 1,406,242 residents . Moreover, for its location, in the core of Europe and its development, Milan is the Italian most important economic centre. The total municipal area is 181.81 km^2^. The city is divided into 88 neighbourhoods, and nine municipalities. The urban structure of Milan significantly changes from the city centre to the peripheral areas. The city developed along radial axes and in concentric circles (the circle of Navigli, the Ramparts, the trolley-bus ring, the railway, the ring road). In more recent decades, Milan experienced positive development through increased international investments, tourism, cultural offerings, and services. Hence, its neighbourhoods and areas are continually changing, renewing its intersections, squares and abandoned areas.

**Fig. 3:**
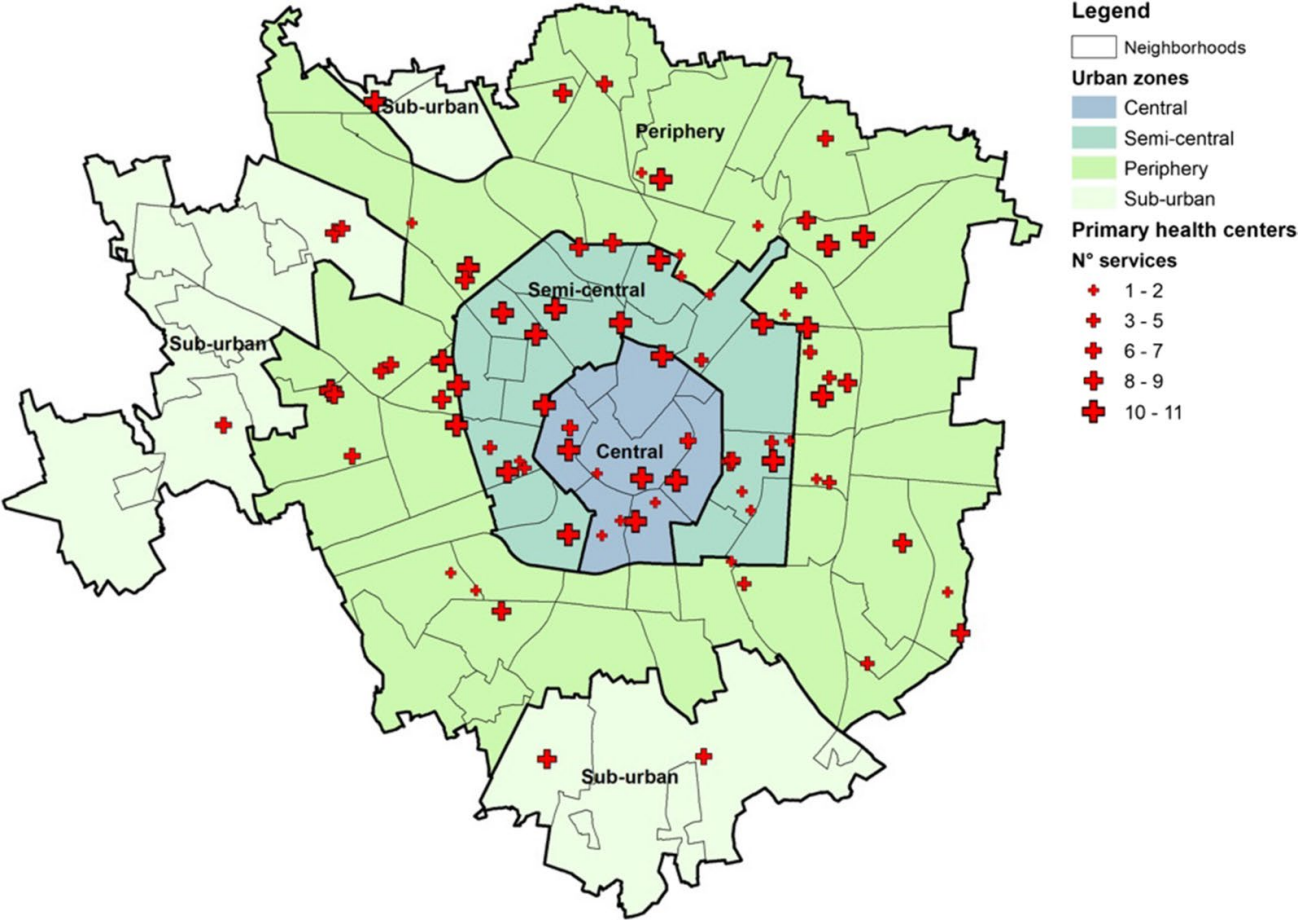
The administrative structure and primary health buildings in the city of Milan

According to the Italian Revenue Agency (Agenzia delle Entrate), the city is divided into four urban zones: Central, Semi-central, Periphery and Sub-urban. The Inland Revenue introduced this territorial division as part of studies concerning real estate values by detecting different areas, based on their geographical location within municipal boundaries, their urban structure, and the prevailing type of building. Figure 3 represents the classification of Milan neighbourhoods according to Agenzia delle Entrate. For this first place, we tried to read and discuss the methodology outputs through this lens which broadly considers both land-use and mobility dynamics.

The map also highlights the localisation and availability of resources for each primary healthcare service, which are the main topic of interest for our application.

Regarding the healthcare provision, the Health Protection Agency of Milan (ATS—Agenzia di Tutela della Salute) is responsible for primary health services for city dwellers, including both public and private centres. The entire system of healthcare supply comprises both public structures and private contracted services: they respectively share 50% of the full demand for healthcare assistance. According to ATS management, public and agreed private services are equal for users, both in terms of performance and costs.

Hence, for the EM2SFCA application, we evaluated the overall accessibility to 11 ambulatory hospitals and 70 neighborhoods ambulatories that serve more than 300,000 elderly people. The available resources of each healthcare structure were addressed as the number of surgeons of interest to the elderly and, consequently, to their health. We select eleven surgeons: cardiology, diabetology, neurology, ophthalmology, orthopaedics, otolaryngology, pulmonology, urology, rehabilitation, dermatology and gynaecology (Table 1).

**Table 1:** The population and ambulatory distribution per urban zones

Urban zones	Total population	Elderly population	Number of ambulatories	Number of surgeons
Sub-urban	121,694	34,125	6	47
Periphery	713,586	178,643	41	256
Semi-central	312,412	73,239	23	164
Central	94,431	22,741	11	73

Around 25% of the total population (308,748 inh.) were aged 65 or above, mainly resident in the semi-central and periphery neighbourhoods. Data come from Italian Statistic Institute.

The analyses are run differently for the three age groups described in Table 2 of the elderly: people aged 65–69 years (young elderlies), 70–74 years (medium elderlies) and 75 and over the years (old elderlies). For the city of Milan, over 58% of the elderly population live in the Periphery neighborhoods and over 24% in the Semi-central neighborhoods. The non-uniform distribution of elderly people in different urban zones is influenced by the various ages of urbanisation and properties’ value. During the development of new peripherical areas, the local authorities did not support the localisation of new urban services useful to ensure good quality of life for the different categories of citizens.

**Table 2:** Elderly people distribution per age group in the urban zones (ISTAT, 2011)

	65–69 years old inhabitants	70–74 years old inhabitants	≥ 75 years old inhabitants	Elderly people (%)
Sub-urban	7789	9432	16,904	11
Periphery	41,307	44,923	92,413	58
Semi-central	17,647	17,950	37,642	24
Central	6090	5665	10,986	7

**Fig. 4:**
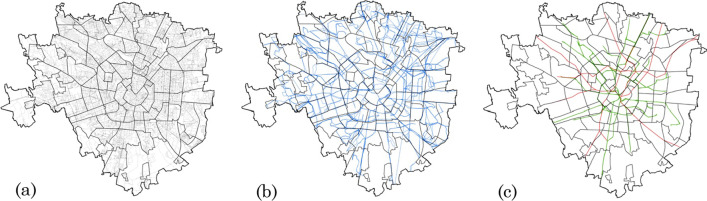
Walking network (**a**), bus network (**b**) and tram and metro network (**c**) in the city of Milano

Concerning the supply side of the transport component, a multimodal network of Milan was created using ArcGIS tools to connect roads shapefile and GTFS (General Transit Feed Specification) data into a network dataset and evaluate travel times. First, OpenStreetMap data was used to create the walking network, considering only pedestrian roads and their slopes (Fig. 4a). Then, GTFS data from the ATM (Azienda Trasporti Milanesi—Milan Transport Company) was used to add bus, tram and metro routes and stops in the transport network (Fig. 4b, c). Since public transport is not a continuous service in space and time, additional modelling operations were needed to connect the pedestrian system to the public transit system. Once the multimodal network was ready, the ArcGIS Network Analyst tool was used to compute an OD (Origin and Destination) matrix, containing in each cell the total travel time to get from a generic hexagonal cell to a certain healthcare centre.

## Results

**Fig. 5:**
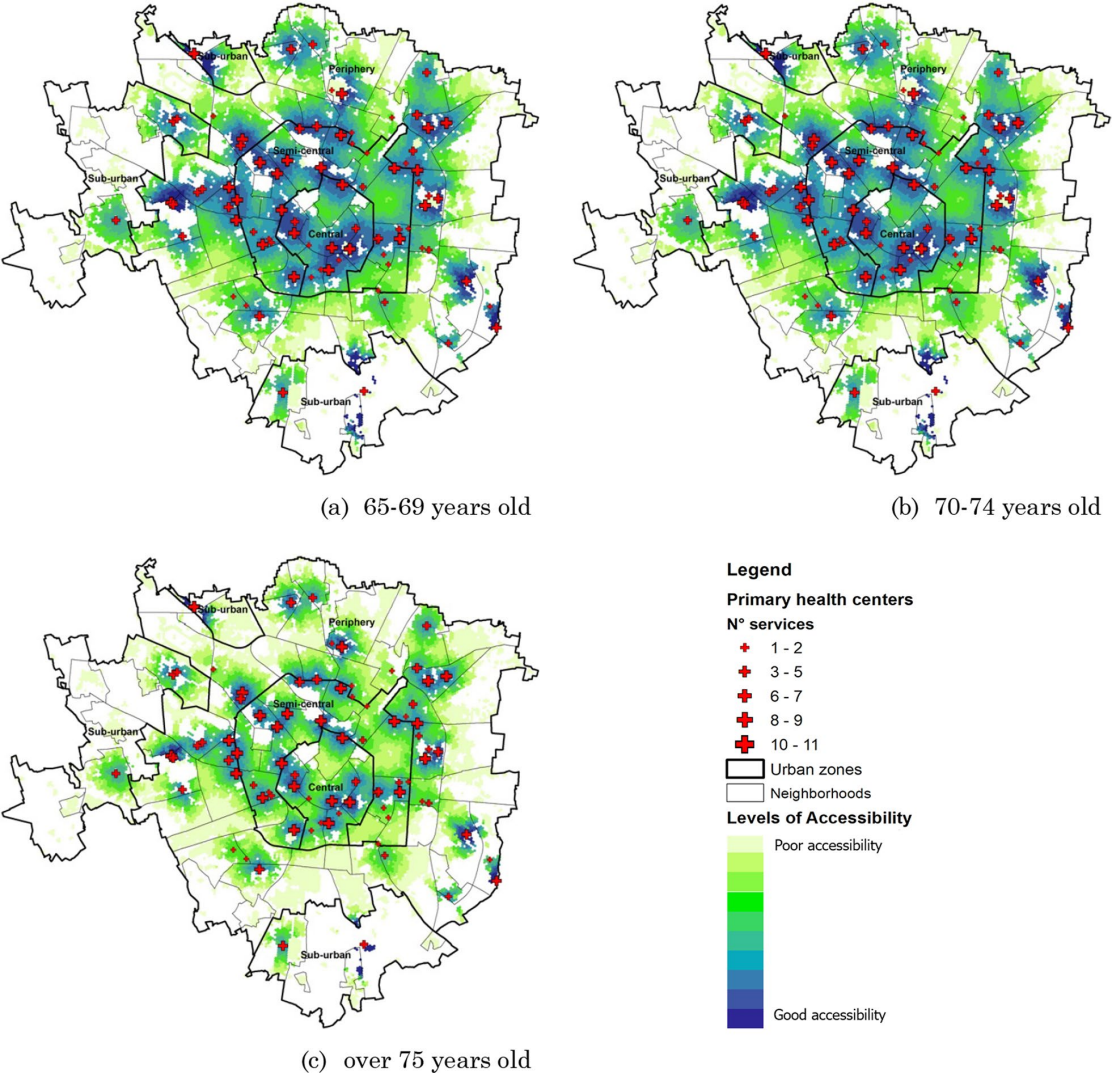
Level of accessibility at the primary health centres in the Milan urban rings for the different elderly age categories

Figure 5 represents the accessibility maps to primary healthcare provision, for each elderly age group in Milan. The EM2SFCA methodology application allows users to locate and quantify people suffering from a poor level of accessibility to essential healthcare services. Elaborations highlight that the elderly dwelling in neighbourhoods farther from the city centre suffers from limited access to such services. Moreover, according to scientific literature hypotheses, it should be noted that people aged 75 and above are more penalised, rather than those aged 65–69, due to their more limited mobility capital.

According to scientifically established and widespread practices (PTAL—Transport for London), ten accessibility levels’ thresholds were chosen though a quantile classification, a data classification method that distributes a set of values into groups containing an equal number of values.

**Fig. 6:**
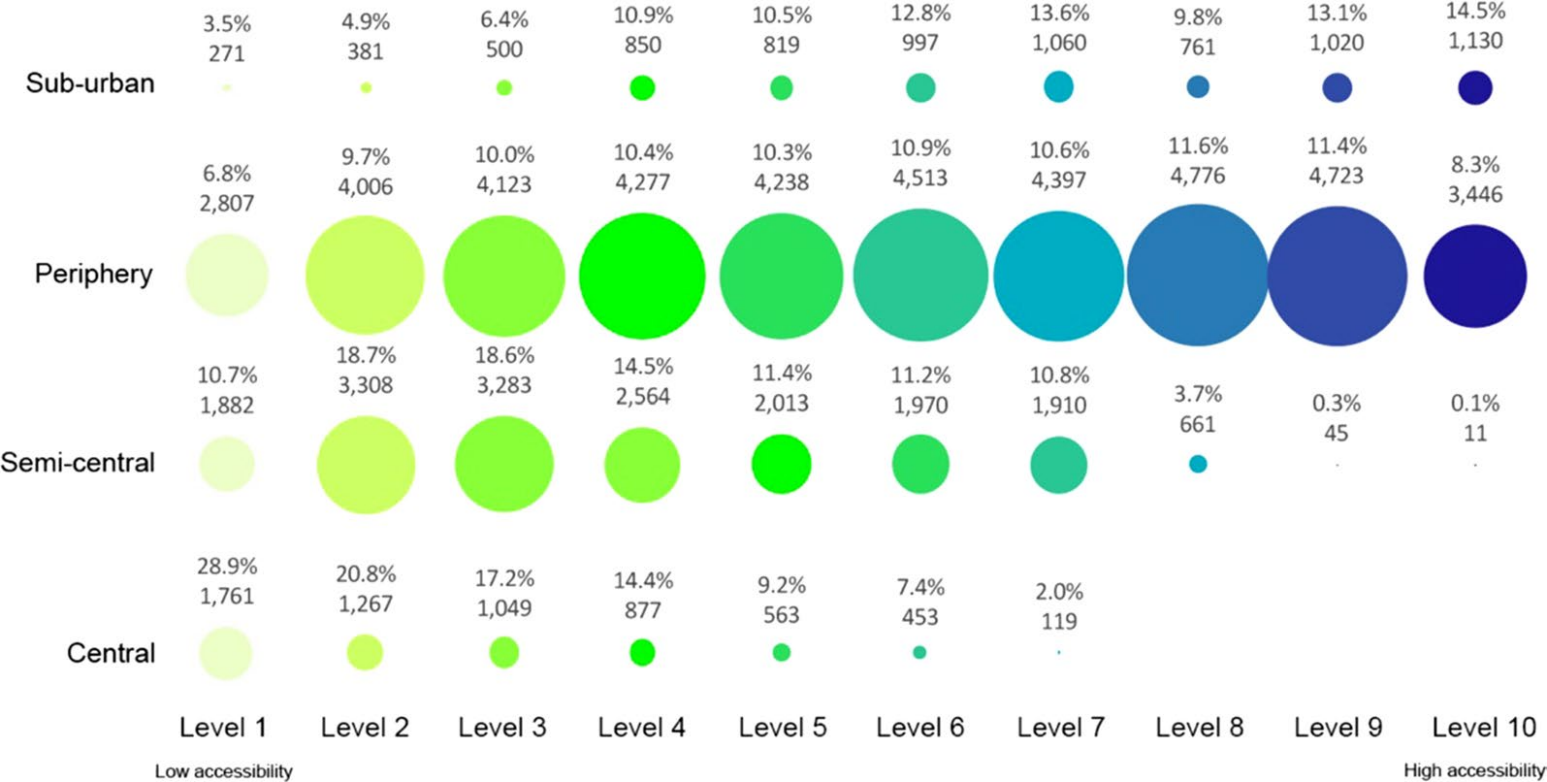
Level of accessibility at the primary health centres in the Milan urban zones for elderly 65–69 years old

**Fig. 7:**
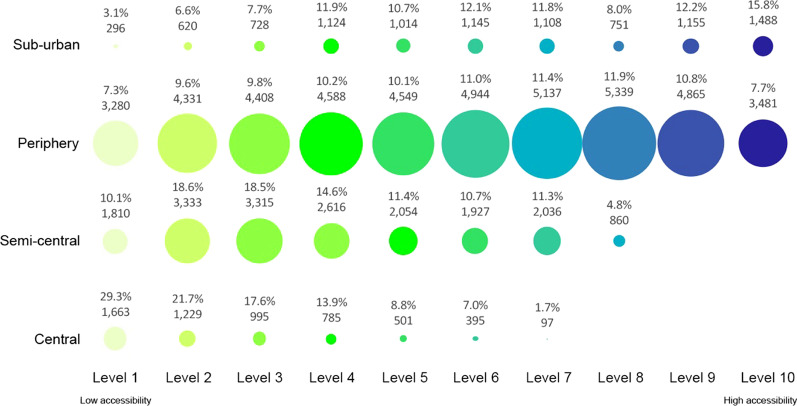
Level of accessibility at the primary health centres in the Milan urban zones for elderly 70–74 years old

**Fig. 8:**
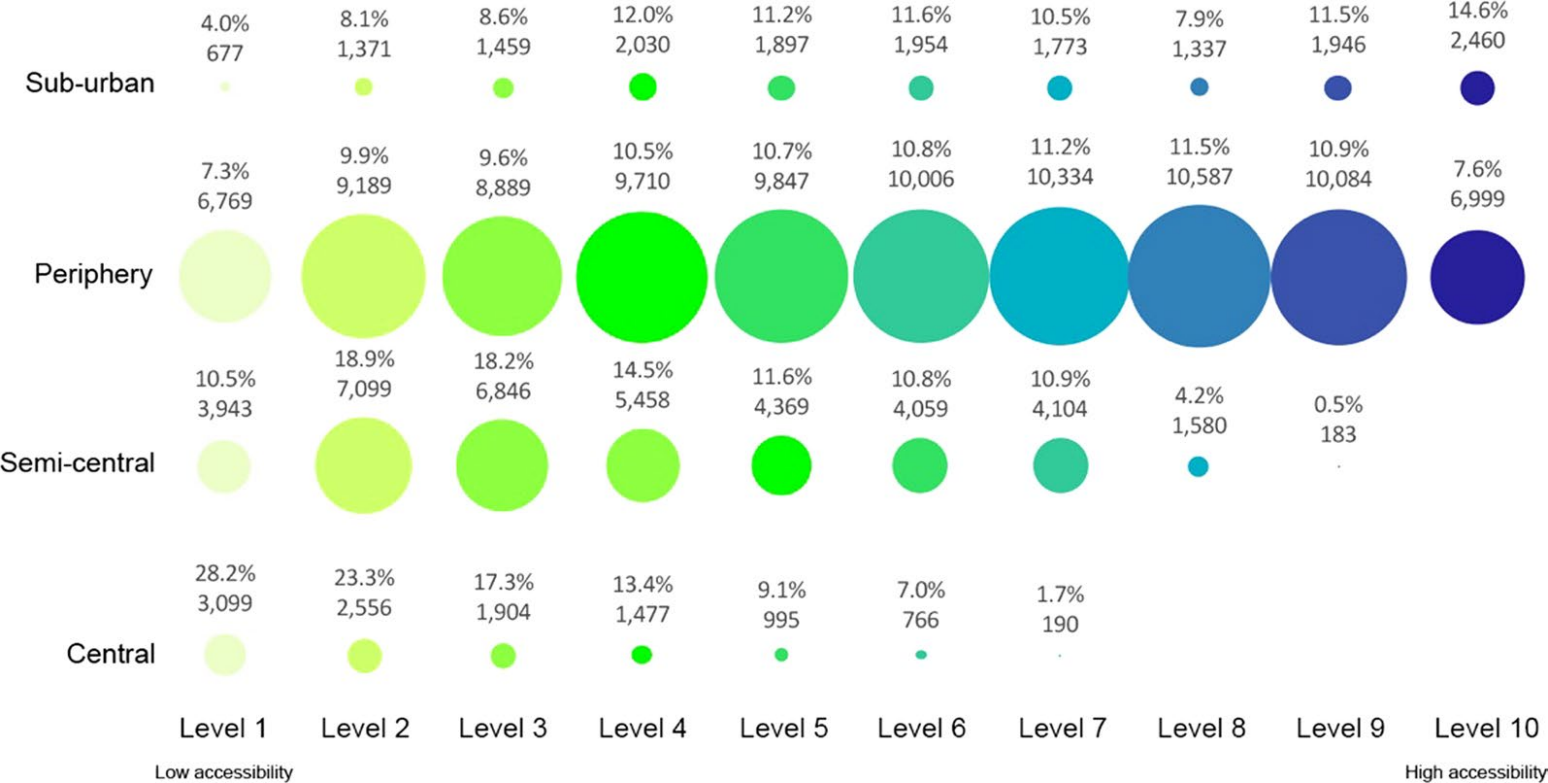
Level of accessibility at the primary health centres in the Milan urban zones for elderly over 75 years old

Figures 6, 7 and 8 show the level of accessibility of elderly people, for every age group, live in each urban area—Central, Semi-Central, Periphery and Sub-Urban.

For the Central neighbourhoods, the results show that over 6500 elderly lives with a low level of accessibility (Level 1) at primary health services, with 28.9% of young elderlies, 29.3% of medium elderlies, and 28.9% of old elderlies.

In the Semi-central zone, the results show that the current localisation of elderly people and the primary health services are not satisfactory. The elderly people who live in this urban zone have low accessibility to primary health services. The numerical results show that over 47% of elderlies are included from level 1 to level 3 of accessibility. The age category with the highest number of people from level 1 to level 3 of accessibility is the older elderlies.

For the Periphery zone, the percentage of elderly people for each level of accessibility does not change significantly in the different age categories. In the neighbourhoods of this urban zone, over 10,000 inhabitants aged 65–69, 12,000 inhabitants aged 70–74, and over 24,000 inhabitants aged over 75 are included from level 1 to level 3 of accessibility (less than 26% of elderlies’ population for the periphery zone). Many neighbourhoods do not have primary health services buildings in this urban zone.

In the Sub-urban, the number of primary health services is low (only six buildings) and located far apart from each other. The results related evidence that the services are located close to a great part of users. Over 35% of elderly people live in areas with accessibility from level 8 to level 10.

**Fig. 9:**
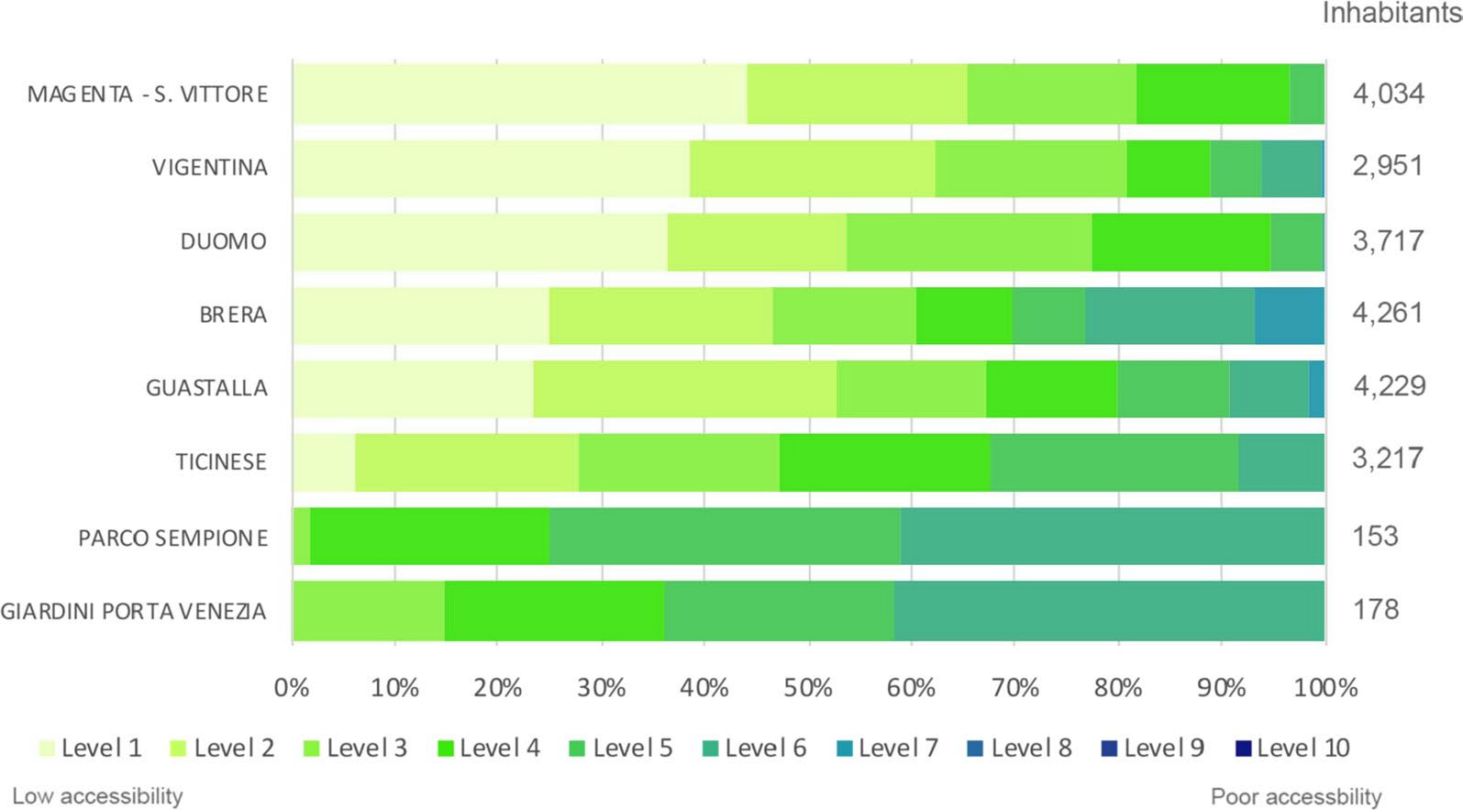
The percentage of elderly people for each level of accessibility at the primary health centres in the Milan Central neighbourhoods

The results illustrated in Fig. 9 evidence the neighbourhoods that need more improvement for the accessibility at primary healthcare for elderly people in the Central urban zone. Considering the number of elderly people that live in the Central urban areas, the most critical neighbourhoods are: Magenta-S. Vittore with less than 3300 older adult inhabitants (81%neighbourhood of total dwellers) from level 1 to 3; Vigentina with around 2300 over 65 inhabitants from level 1 to 3; Duomo with all older adults (around 3700) from the level 1 to 6. It is unnecessary to consider Parco Sempione and Giardini Porta Venezia neighbourhoods because they host only 332 over 65 inhabitants.

**Fig. 10:**
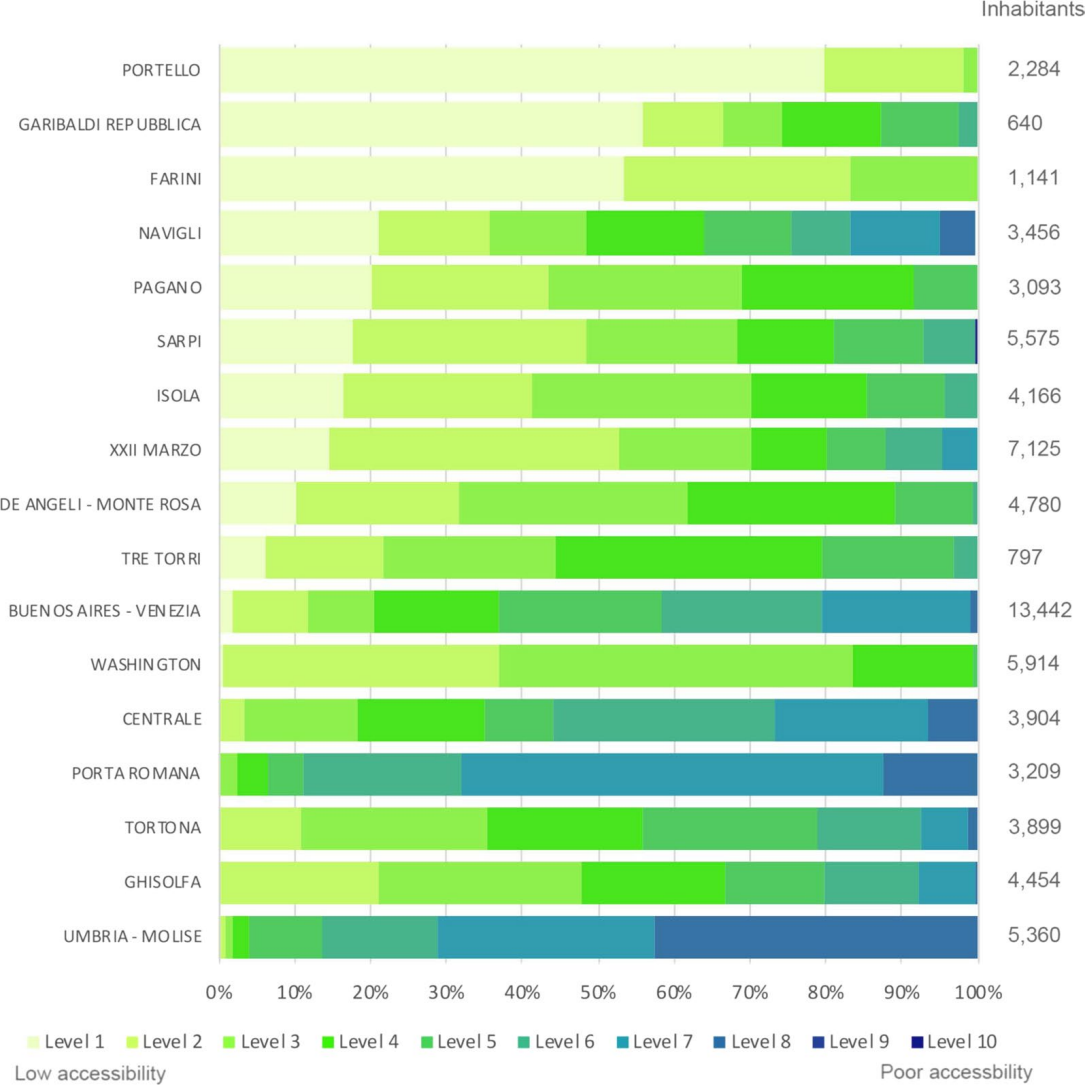
The percentage of elderly people for each level of accessibility at the primary health centres in the Milan Semi-central neighbourhoods

The results illustrated in Fig. 10 evidence the neighbourhoods with the lowest accessibility at primary healthcare for elderly people in the Semi-central urban zone. The most critical neighbourhoods in this urban zone, considering the population located, are Portello (2284 elderlies from level 1 to 3); Pagano (3093 elderlies from level 1 to 5); Sarpi (5576 elderlies from level 1 to 6); XXII Marzo (over 5000 elderlies from level 1 to 3); Washington (over 4900 elderlies from level 1 to 3).

Although the methodology has some limitations, due to the unavailability of more recent and reliable data (since population data refers to the last national census run in 2011) and the inability to calibrate distance-decay functions on actual mobility attitudes and travel choices of the elderly in Milan, EM2SFCA shows several interesting issues. First, it has proven its ability to evaluate urban accessibility and, hence, support policymakers in managing urban services in cities, such as healthcare ones, through the accessibility lens. Testing different operating scenarios, both from transport- and land-use-sides, the methodology can suggest strategies and actions to increase urban resilience which, if properly integrated in urban planning tools, can guarantee a good level of accessibility to essential urban services, both for the elderly and for city dwellers in general with limited mobility. Moreover, since the method considers supply-side features, it can help optimise both location and distribution of existing resources.

## Discussion and conclusions

This paper presents a methodology aiming at identifying urban areas with poor accessibility to healthcare provision services, for the perspective of ageing population. The focus of the paper is not only how to compute urban accessibility for welfare purpose but also to develop a method to support decision-makers to identify where and how to invest (e.g., in service localisation or management? public transport or walkable roads?) in order to improve the accessibility to a selected number of healthcare services.

The state of the art has shown that while there is a large body of literature focusing on mobility and needs of older adults, accessibility measures are rarely applied to drive urban planning policies [46, 47], as also pointed out by World Health Organization (WHO) report on measuring the age-friendliness of cities (2015). The methodology has been applied to one of the most important Italian metropolitan cities, Milan, after being tested in other urban contexts [34, 35]. This proves that accessibility-based methodology can be efficiently applied on the large urban scale to identify areas to be further developed and where to invest to limit age and, more in general, social inequalities.

Moreover, the paper shows the potential and the usability of the accessibility concept in urban planning and confirm that accessibility instruments are applicable in practice when they are included in a framework easy to be computed by technicians and interpreted by decision-makers.

Scientific research demonstrates that the evaluation of urban accessibility can support urban planners in the localisation and integration of different urban services. Hence, it shows on one hand that accessibility-oriented planning practices could be effective to improve the quality of life according to heterogeneous dwellers’ needs and capabilities. On the other, standard practices in urban planning lack some insight on the accessibility issue because of noticeable critical aspects—namely, from the large amount of data needed to the use of spatial analysis instruments and their interpretability. Decision-makers and technicians may find accessibility measures and their potentialities challenging to understand and manage. Furthermore, structures, offices, or technicians that work to define common strategies for transport and land-use planning are missing from territorial planning authorities. This gap in planning practices is one of the main causes of accessibility issues for urban services, especially for the most vulnerable citizens.

During the following years, the continuous growth of the proportion of elderly people in developed countries will increase the demand for services, especially in light of the Covid-19 outbreak and consequent vulnerability of the elderly [34, 35, 48]. Hence, urban services need to be appropriately designed: structures’ locations, services, the transport system supply, and the urban morphology are some of the main features influencing users’ accessibility to urban services.

This research proposes an EM2SFCA—Elderly-oriented Multimodal 2 Steps Floating Catchment Area—methodology that considers land-use and transport features, both from the supply and demand side, given that urban accessibility depends on the balance between these two opposite aspects of the urban environment. Moreover, since the methodology was designed to assess urban accessibility from the elderly perspective, behavioural elements are essential to understand their limited mobility capital better. The method has been designed in GIS environment since it is an unrivalled framework for gathering, managing, and analysing data. However, from the operational viewpoint, EM2SFCA presents some difficulties: in fact, it requires the estimation of some parameters, such as the distribution of population within regular cells, and the collection of data and information, such as, for instance, the localisation and availability of healthcare services, both public and private.

The method has been applied to a significant case study, the city of Milan since it reflects the ageing trends of whole Europe. The results of the analyses carried out suggest that more that 50% of elderly people living in peripheral and suburban neighbourhoods suffer for poor accessibility to healthcare services. This share is completely upset for central urban areas.

The results of the application have allowed to evaluate accessibility in current working scenario, both from the transport and land-use sides and to identify priority areas of intervention. Further applications of the model, based on different scenarios, would suggest the most suitable strategies and actions, which could involve re-organization of healthcare and public transit services, as well as the quality of walkable network. Moreover, he results of the methodology have proved a good depiction of the main accessibility components. For what concerns the land-use component, the city-wide hexagonal grid and preliminary analyses for the supply of urban services and their resources provide a detailed representation of urban territory and its activity system. Then, the computation of actual travel times, thanks to GTFS data, provides a high-quality portrayal of urban mobility system opportunities (walking and transit networks). Finally, the use of distance decay functions as well as decreasing walking speeds for older adults depicts the individual behaviour of elderly people.

In terms of takeaway for practice from this academic experience, the planning of healthcare services in urban areas—particularly for the elderly who are the most vulnerable users, as Covid-19 has highlighted—should follow two basic guidelines. The first concerns the allocation of resources within cities which should take into account the varied socio-economic backgrounds of different groups. Moreover, as noted in previous paragraphs, studies have shown that elderly people suffer from poor accessibility to essential services due to the lack of mobility capital. Hence, the second guideline involves improvements for the mobility system. The availability of multiple travel choices is crucial to satisfy personal and social needs, especially for those who have a limited mobility capital as older adults.

Hence, age-friendly strategies are needed and, in general, policymakers should guarantee equal accessibility to a wide range of urban services for every citizen, no matter their social, economic and physical conditions.

Thus, our research has some limitations. Due to the lack of available data, the distance-decay functions do not consider older people’s behaviours for a Milan context; these were deduced from literature. Second, we integrated two transport modes, pedestrian and public, but further modes could be considered, such as private modes. The availability of sustainable mobility facilities could be considered, as well. Finally, for these reasons, we would further develop the EM2SFCA method to investigate its potential effectiveness in urban planning strategies. For example, we would look at the relative benefits of (1) validating the procedure within other urban contexts; (2) considering the urban and environmental aspects that influence the walkability of the elderly in different times of the day, in different seasons and different social-distancing scenarios; (3) evaluating the optimal location of new transport and health facilities to improve the urban accessibility level of under-served areas; (4) extending the 2SFCA methodology to other age groups, by calibrating ad hoc distance decay function for heterogeneous mobility capitals; (5) developing a web-server GIS tool to simplify the application of the procedure and the interpretability of its results.

## Data Availability

Not applicable.
